# Code and data from an ADALINE network trained with the RTRL and LMS algorithms for an MPPT controller in a photovoltaic system

**DOI:** 10.1016/j.dib.2020.106296

**Published:** 2020-09-08

**Authors:** Julie Viloria-Porto, Carlos Robles-Algarín, Diego Restrepo-Leal

**Affiliations:** Universidad del Magdalena, Facultad de Ingeniería, Carrera 32 No 22 – 08, Santa Marta, Colombia

**Keywords:** ADALINE, Dynamic neural network controller, MPPT controller, LMS algorithm, PV module, RTRL algorithm

## Abstract

This paper presents a detailed description of the data obtained as a result of the computational simulations and experimental tests of an MPPT controller based on an ADALINE artificial neural network with FIR architecture, trained with the RTRL and LMS algorithms that were used as mechanisms of control in an off-grid photovoltaic system. In addition to the data obtained with the neural control method, the data for the MPPT controller based on the traditional Perturb and Observe (P&O) algorithm are presented. The simulations were performed in MATLAB/Simulink software without using the Neural Network Toolbox for controller training. The experimental tests were performed in an open space without shaded areas, exposing the neurocontroller to varying environmental conditions. Additionally, the scripts developed in MATLAB for the neural training algorithms used in the simulations are presented. These computational simulations were structured in five test cases to represent the behavior of each controller under varying environmental conditions. The codes developed in C are part of the implementation of the MPPT neurocontroller in the PIC18F2550, from which the experimental data were obtained. The data and codes presented in this research are available in the Mendeley Data repository, which allows evaluating the performance and optimizing the training algorithms with the purpose of improving the control methods applied to photovoltaic systems.

## Specifications Table

**Table:** 

Subject	Computer Science
Specific subject area	Artificial Intelligence, Off-Grid Photovoltaic Systems
Type of data	Table Figure MATLAB code C code
How data were acquired	The simulation data were obtained using MATLAB/Simulink R2017a. The voltage and current values used as input data for the Adaptive Linear Neuron (ADALINE) network were obtained by computer simulations of a 65 W photovoltaic (PV) module. For the Real-Time Recurrent Learning Gradient (RTRL) and iterative Least Mean Square (LMS) algorithms, only six (6) of those data were needed for the neural network training. In the case of the standard LMS algorithm, only three (3) data were used for training the neural network. The experimental data were obtained with the following devices: PIC18F2550 Microcontroller, UNI T UT33C+ Digital Multimeter, FLUKE 116 Multimeter, Solar radiation pyrometer (Dr. Meter SM-206). During the experimental tests, the voltage and current values were acquired at the connection between the PV module and the neural controller. These elements, together with the dc-dc converter and a 12 V battery, were exposed to varying environmental conditions (rainy and sunny days).
Data format	Raw Analyzed Filtered
Parameters for data collection	The mathematical model of the photovoltaic module (Yingli Solar JS 65), the ADALINE network architecture and the RTRL and LMS training algorithms were designed and implemented in MATLAB/Simulink R2017a, without using any of the toolboxes available for this type of modeling. The computational simulations were performed on 2 computer equipment in order to measure the performance of each training algorithm and evaluate the response of the PV system: 1) HP laptop with Windows 10 Pro 64-bit, Intel Core i5–5300 vPro CPU @ 2.30 GHz and 8GB of RAM, 2) Dell laptop, with Windows 10 pro 64-bit, Intel Core i7–7500 U CPU @ 2.70 GHz and 8GB of RAM. The C code, designed for experimental testing of the MPPT control system, was developed, debugged, and compiled with the tools of the MPLAB XC8 compiler. Subsequently, it was integrated into the MPLAB X IDE desktop environment. Finally, the implementation was performed on a PIC18F2550 microcontroller.
Description of data collection	The data used as input parameters for the MPPT control system were obtained from the mathematical modeling of a PV module, which was implemented using Simulink block diagrams to simulate physical and electrical behavior. The input data for the PV module model are solar irradiance and surface temperature. These data were generated with the signal builder block of Simulink, which was manually edited to provide irradiance values between 0 W/m^2^ and 1000 W/m^2^ and temperature between 0 °C and 100 °C, to represent the variability in environmental conditions. The PV module output data are the voltage and current values, which were used as input parameters for the dc-dc converter and MPPT controller. The power data, obtained with the algebraic operation between the voltage and the current, were normalized to subsequently be used in the neural network and the training algorithms. To obtain the experimental data from the controller, work sessions were performed for 3 consecutive days. During the tests, the solar irradiance and surface temperature values of the PV module were in the ranges of 56 W/m^2^ - 1030 W/m^2^ and 28.7 °C - 50 °C respectively. The output power was supplied to the neural controller and the dc-dc converter. These power data were processed and normalized before entering the MPPT control device.
Data source location	Institution: Universidad del Magdalena City/Town/Region: Santa Marta, Magdalena Country: Colombia Latitude and longitude (and GPS coordinates) for collected samples/data: 11°13′32.9″N 74°11′12.9″W
Data accessibility	Repository name: Supplementary material: Code and data from an ADALINE network trained with the RTRL and LMS algorithms for an MPPT controller in a photovoltaic system Data identification number: 10.17632/mbt6845jjb.1 Direct URL to data: https://data.mendeley.com/datasets/mbt6845jjb/1
Related research article	Viloria-Porto, J., Robles-Algarín, C., Restrepo-Leal, D., A Novel Approach for an MPPT Controller Based on the ADALINE Network Trained with the RTRL Algorithm, https://doi.org/10.3390/en11123407[1].

## Value of the Data

● With MATLAB scripts, researchers can recreate the behavior of the ADALINE network with the RTRL and LMS training algorithms [1]. With these programs, it is possible to understand the operation of the ADALINE network and its application in PV systems.
● With the code provided in C language, it is possible to implement the MPPT controller based on the ADALINE network with the RTRL training algorithm on a microcontroller. Thus, researchers can perform the calculation of parameters such as efficiency, computational time and computational cost; in order to make comparisons with other controllers reported in the literature.
● These data are useful to other researchers interested in designing and implementing MPPT controllers for practical applications in off-grid photovoltaic systems.
  With this data, readers can evaluate the behavior of PV systems under sudden changes in operating conditions. It is possible to understand the performance of PV modules for abrupt changes in solar irradiance and operating temperature.
● These data are useful for making comparisons with other algorithms with high computational efficiency [2]. Similarly, comparisons can be made with traditional algorithms used to track the maximum power point of PV modules, such as the P&O algorithm and the incremental conductance method.

## 1 Data Description

This paper presents the codes developed and the data obtained as a result of the simulation and implementation of a PV system composed of a PV module, an MPPT controller based on an artificial neural network, a dc-dc converter and a battery used as load. This PV system was exposed to various configurations, as detailed below.

### 1.1 Input data used in the simulation of the PV system

Changes in environmental conditions for the solar irradiance and temperature were generated in the Signal Builder block of MATLAB/Simulink [3], starting from a seed to generate pseudo-random values. This way, five simulation scenarios were established, represented by the changes in the input variables of the PV module. These scenarios and the input data used for each of them are described below.

Case 1: Standard test conditions with a solar irradiance of 1000 W/m^2^ and temperature of 25 °C. These data can be found in the files: Irradiance_Case1.csv and Temperature_Case1.csv, in the following folder: Data/Computational_Tests/ of the supplementary material.

Case 2: Variable irradiance and constant temperature of 25 °C with a simulation time of 1 s. See in the supplementary material: Irradiance_Case2.csv and Temperature_Case2.csv. Folder: Data/Computational_Tests/.

Case 3: Constant irradiance of 1000 W/m^2^ and variable temperature, with 3 s of simulation time. See in the supplementary material: Irradiance_Case3.csv and Temperature_Case3.csv. Folder: Data/Computational_Tests/.

Case 4: Scenario with variable values for solar irradiance and temperature, during 1 s of simulation. See in the supplementary material: Irradiance_Case4.csv and Temperature_Case4.csv. Folder: Data/Computational_Tests/.

Case 5: Slightly staggered irradiance with subsequent sudden upward and downward changes, while, for a simulation time of 12 s, the temperature remains at a constant value of 25 °C. Then, the temperature changes abruptly to complete the 25 s of simulation. See in the supplementary material: Irradiance_Case5.csv and Temperature_Case5.csv. Folder: Data/Computational_Tests/.

These input signals can be easily viewed by running the Python script available in Codes/Read.py of the supplemental material.

### 1.2 PV module model

The PV module used in the computational simulations was modeled using Eq. (1) [4,5].

(1) $$I\left(V\right)=\frac{I_{x}}{1-e^{\left(\frac{-1}{b}\right)}}\left[1-e^{\left(\frac{V}{bV_{x}}-\frac{1}{b}\right)}\right]$$

Where the open circuit voltage V_x_ and the short circuit current I_x_ are defined by Eqs. (2) and (3).

(2) $$V_{x}=s\frac{E_{i}}{E_{iN}}TC_{V}\left(T-T_{N}\right)+sV_{\text{max}}-s\left(V_{\text{max}}-V_{\text{min}}\right)e^{\left(\frac{E_{i}}{E_{iN}}\ln\left|\frac{V_{\text{max}}-V_{oc}}{V_{\text{max}}-V_{\text{min}}}\right|\right)}$$

(3) $$I_{x}=p\frac{E_{i}}{E_{iN}}\left[I_{sc}+TC_{i}\left(T-T_{N}\right)\right]$$

The data obtained from the behavior of the PV module for each of the computational test cases described above can be consulted in the folder: Data/Computational_Tests of the supplementary material.

Case 1: PVModule_Case1.csv

Case 2: PVModule_Case2.csv

Case 3: PVModule_Case3.csv

Case 4: PVModule_Case4.csv

Case 5: PVModule_Case5.csv

All files have two columns: Time (s), which contains the simulation time in seconds and PV Module Power (W), which represents the power in watts, obtained by the PV module when it is exposed to the conditions of the test cases.

### 1.3 MPPT controller

The data obtained with four control methods for the test scenarios described above are presented. The first was the traditional Perturb and Observe (P&O) method and the others are based on an ADALINE artificial neural network [6] with three training algorithms, the RTRL and the LMS in their iterative and standard versions [1,6].

The function of these controllers is to allow the highest power transfer between the PV module and the battery. These control methods receive as an input signal the power obtained from the PV module and their output is a PWM signal. The data obtained with all the drivers is stored in the folder: Data/Computational_Tests/ of the supplementary material.

• P&O method: The files for this controller have two columns of data, Time (s) containing the time in seconds and P&O Power (W), which includes the power obtained with this control method. Thus, for test cases 1, 2, 3, 4 and 5, the data obtained are available in the csv files of the supplementary material: P&O_Case1, P&O_Case2, P&O_Case3, P&O_Case4 and P&O_Case5.
• ADALINE network with the Iterative LMS algorithm: The power extracted by this controller in each test case is contained in the csv files: IterativeLMS_Case1, IterativeLMS_Case2, IterativeLMS_Case3, IterativeLMS_Case4 and IterativeLMS_Case5, which contain two columns with the time data in seconds and power in watts (Time (s) and Iterative LMS Power (W)). The implementation of the ADALINE network and the iterative LMS training algorithm can be found in the MATLAB script named training.m, available in the folder Codes/ of the supplementary material.
• ADALINE network with the Standard LMS algorithm: The files in csv format: LMS_Case1, LMS_Case2, LMS_Case3, LMS_Case4 and LMS_Case5, represent the behavior of the MPPT controller based on the ADALINE neural network with the standard LMS training algorithm. These files correspond to the five test cases and contain the data for the time and power output (Time (s) and LMS Power (W)). In the file: Codes/trainingLMS.m of the supplementary material, the implementation of the ADALINE network with the standard LMS training algorithm is presented.
• ADALINE network with RTRL algorithm: The power that this MPPT controller can extract from the PV system in the 5 test cases, are found in the csv files in the folder Computational_Tests/ of the supplemental material: RTRL_Case1, RTRL_Case2, RTRL_Case3, RTRL_Case4 and RTRL_Case5. These files are made up of two columns: Time (s), which contains the time in seconds and RTRL Power (W), which has the power extracted by this controller. In the file: trainingRTRL.m, available in the folder Codes/ of the supplementary material, the implementation of the ADALINE network with the RTRL training algorithm is presented.

### 1.4 Experimental data

The experimental stage was performed for 3 consecutive days in a completely open space without shaded areas. The files that were generated during the tests contain the power of the PV module and the power output of the dc-dc converter, separating each power data by a comma. See in the folder of the supplementary material: Data/Experimental_Tests, the files in txt format Session1, Session2, Session3.1, Session3.2 and Session3.3. It should be noted that in the experimental stage only the neuronal controller with the RTRL algorithm was implemented.

These data were stored using the serial communication integrated into the MPPT controller. The C language source code for the implementation of the MPPT controller based on the ADALINE network with the RTRL training algorithm was designed for the PIC18F2550 microcontroller. Therefore, the files: main.c, fuses.h, EUSART.h, LCD_Nokia.h are presented in the folder Codes/ of the supplementary material. The Processing script to graph the data obtained is contained in the file Codes/TerminalSerie.pde. It is possible to observe these signals after being stored in txt files using the script developed in MATLAB: Codes/ProcessingTXT.m.

**Fig. 1:**
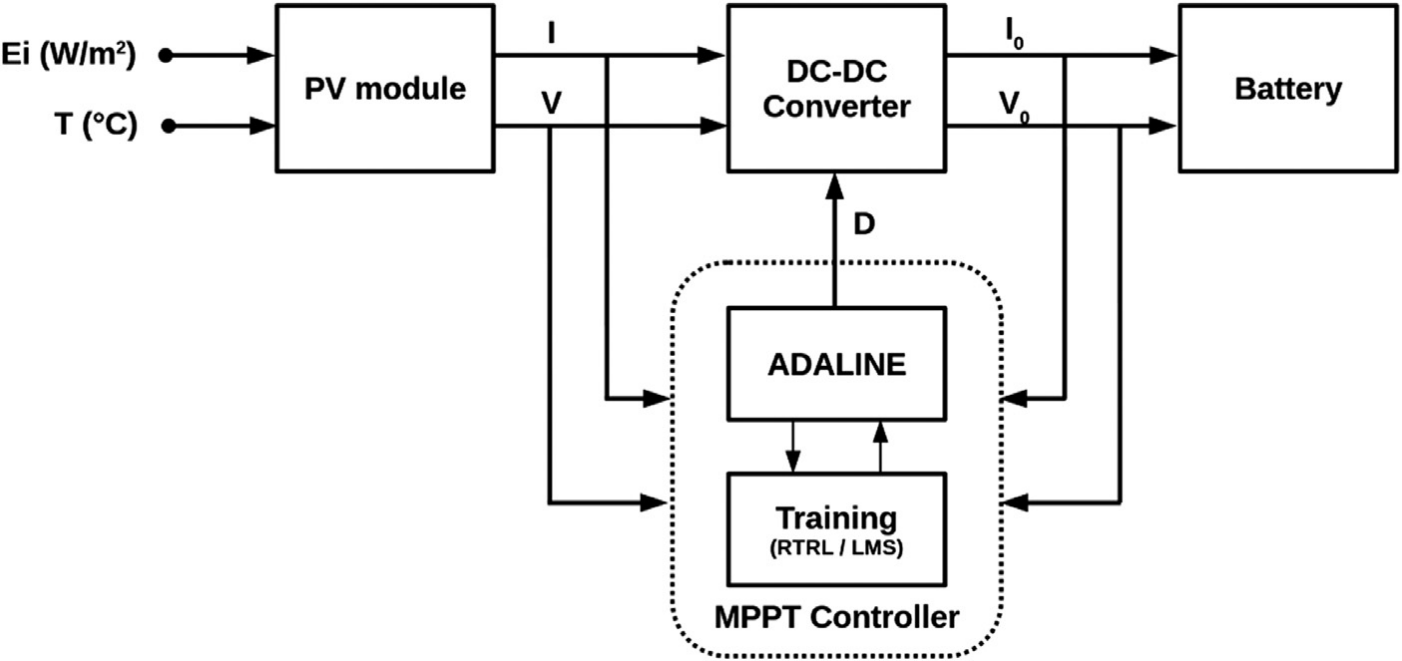
Photovoltaic system with the MPPT controller based on the ADALINE network.

**Fig. 2:**
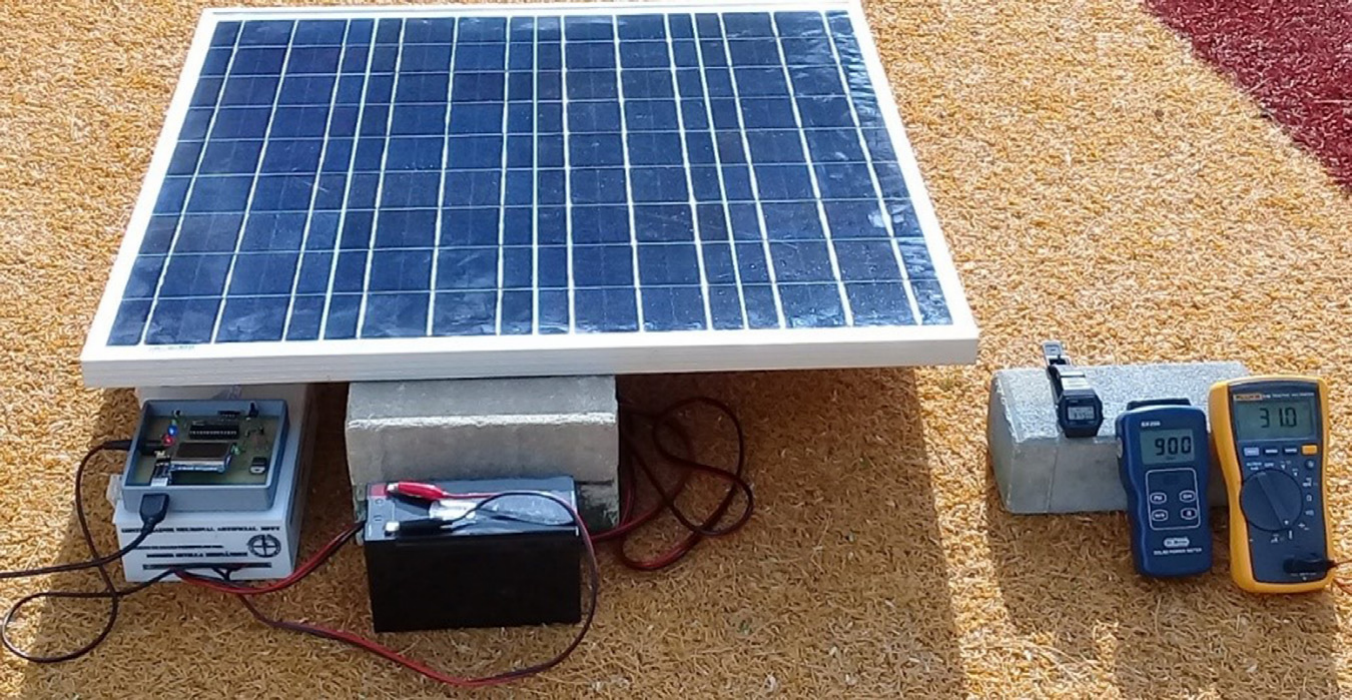
Experimental tests with the photovoltaic system.

Fig. 1 shows the general scheme implemented for the simulations and the experimental stage. The electrical characteristics of the PV module used are shown in Table 1. Finally, Fig. 2 shows the system with all the components used in the experimental section.

**Table 1:** Electrical parameters at STC.

Electrical parameters	Value
Power output (P_max_)	65 W
Power output tolerances (ΔP_max_)	± 5%
Open-circuit voltage (V_oc_)	21.70 V
Short-Circuit current (I_sc_)	4 A
Voltage at P_max_	17.50 V
Current at P_max_	3.71 A
Module efficiency (ɳ_m_)	12.80%

## 2 Experimental Design, Materials, and Methods

The acquisition of simulation and experimental data was performed according to the block diagram presented in Fig. 1.

In the case of the simulation data, the voltage and current values were obtained from the mathematical modeling of the PV module using the blocks available in the MATLAB/Simulink software. The electrical parameters used in the PV module model correspond to the standard test conditions (STC): solar irradiance (E_i_) of 1000 W/m^2^ and temperature of 25 °C. Table 1 shows the other electrical parameters used for modeling the PV module.

As shown in Fig. 1, the PV module model receives solar irradiance and surface temperature as input signals. Once the behavior of the PV module is simulated, the voltage and current signals are injected to the MPPT controller and dc-dc converter models. The data entering the MPPT controller are algebraically operated to obtain the power delivered by the PV module. Subsequently, these power values are normalized.

The dc-dc converter block, which receives the signals from the PV module, also receives the PWM signal generated by the MPPT controller with the training algorithms (RTRL, Iterative LMS, Standard LMS). This PWM signal does not require impedance coupling since the interconnection between these blocks is performed in computer simulations. This way, the power obtained from the converter can be delivered directly to the 12 V battery.

After obtaining the simulation data, the implementation of the PV system was performed to obtain the experimental data. It should be noted that in this case only the controller with the RTRL algorithm was implemented. This experimental process was performed in an uncontrolled environment, with the purpose of exposing the PV system to real environmental conditions.

For the tests, a YL65P-17b Yingli Solar module was used, as shown in Fig. 2. The ACS712 of 30 A Hall effect sensor was used to measure the current, while the E3-01M module was used to measure the voltage. These sensors are built into the dc-dc converter block and allow voltage and current signals to be delivered to the neural controller.

The power values obtained are normalized and processed by the neural controller, which tracks the maximum power point of the PV module to adjust the set point and generate a PWM signal. This PWM signal controls the output of the converter to regulate the electrical energy that is delivered to the 12 V battery. The data obtained during the experimental tests were stored in text files.

In the text files, the input power values that describe the experimental behavior of the PV module, and the output power values obtained after the action of the dc-dc converter and the neural controller were stored. These data can be plotted when executing the script developed in MATLAB (See Codes/ProcessingTXT.m in the supplementary material).

## Declaration of Competing Interest

The authors declare that they have no known competing financial interests or personal relationships which have, or could be perceived to have, influenced the work reported in this article.

## Appendix Supplementary materials

Image, application 1

Image, application 2

Image, application 3
